# The term "carcinoid" is a misnomer: the evidence based on local invasion

**DOI:** 10.1186/1756-9966-28-15

**Published:** 2009-02-10

**Authors:** Jun Soga

**Affiliations:** 1 Niigata Seiryo University, Niigata, Japan

## Abstract

**Background:**

Since Oberndorfer proposed the term "carcinoid" in 1907, over 100 years have passed. This attractive term was initially proposed for 6 cases of his own experience with 12 submucosal lesions in the small intestine.

Oberndorfer summarized the characteristic features of these lesions as follows: (1) small in size and often multiple, (2) histologically undifferentiated with a suggestion of gland-formation, (3) well-defined without any tendency to infiltrate the surroundings, (4) no metastases, and (5) apparently slow-growing reaching no significant size with a seemingly harmless nature.

**Review:**

This article stresses the malignant nature of "carcinoid" on the basis of local invasion prior to metastases in the first two sessions, (1) with Oberndorfer's original diagram, and (2) with an experimental observation on extraglandular microcarcinoid in a form of "budding".

Next, (3) a statistical comparison between a carcinoid group and a non-carcinoid ordinary carcinoma group is introduced on metastasis rates at an early stage with two prescribed factors of the depth of invasion restricted within the submucosa (sm-lesion) and a small tumor size category of 1 cm to 2 cm: the carcinoid group exhibited metastasis rates higher than those in the ordinary carcinoma group when calculated in the stomach and rectum.

In the author's experience, "carcinoids" are malignant not only in the gastrointestinal tract but also in the other sites on the basis of local invasion.

Lastly, (4) discussion on the terminology of "carcinoid" as a misnomer is carried out.

Adequate terms referring to the entity of this malignant tumor group are discussed. One of the most adequate and brief terms for "carcinoid" that is included now in neuroendocrine tumor group would be "endocrinocarcinoma" as per the author's proposal, followed by NEC (neuroendocrinocarcinoma) or GEC (gut endocrinocarcinoma).

**Conclusion:**

The term "carcinoid" is a misnomer that can be confirmed on the basis of local invasion prior to metastases. "No metastases without local invasion" is not of a negligible importance.

## Background

Since Oberndorfer proposed the term "carcinoid" in 1907, over 100 years have passed. This attractive term was initially used for 6 cases of his own experience with 12 submucosal lesions in the small intestine.

Oberndorfer summarized the characteristic features of these lesions as follows: (1) small in size and often multiple, (2) histologically undifferentiated with a suggestion of gland-formation, (3) well-defined without any tendency to infiltrate the surroundings, (4) no metastases, and (5) apparently slow-growing reaching no significant size with a seemingly harmless nature.

## Review

### Introduction

In this short article, the malignancy of carcinoids is stressed on the basis of local invasion prior to metastase in the first two sessions.

A statistical comparison of metastasis rates between a carcinoid group and a non-carcinoid ordinary carcinoma group is introduced at an early stage with two prescribed factors of the depth of invasion and a small tumor size category.

Finally, the terminology of carcinoid as a misnomer is discussed.

### Reevaluation of Oberndorfer's original diagram of "submucosal nodule"

Characteristic features of lesions described by Oberndorfer are well reflected in a beautiful and precise diagram in Fig. 1 in his article [1], indicating a lesion involving a small portion of the mucosa and a large space of the submucosa, the latter seemingly well-defined but without encapsulation.

The findings of this lesion indicate, however, apparent malignancy of the tumor with the small original site in the mucosa invading down continuously into the submucosa forming a larger submucosal nodule as a result. Thus, the lesion is a malignant epithelial tumor, namely a carcinoma, but not a "carcinoma-like" tumor of benign nature that was initially described as a carcinoid.

### Extraglandular microcarcinoid in a form of "budding"

All gastrointestinal "carcinoids" are malignant at their very beginning, "budding" stage, of neoplastic formation.

The early developmental process of carcinoid formation may be hypothetically divided into three stages as shown in Table 1.

**Table 1:** Microproliferation of argyrophil cells [2]

I		Hyperplastic: Intraglandular
II		Preneoplastic: Intraglandular
III		Neoplastic
	IIIa	Intraglandular
	IIIb	Extraglandular ("budding": microinvasion)

Extraglandular neoplastic formation starts with a form of "budding" (IIIB).

An observation on consecutive serial sections of the glandular stomach of an experimental animal clearly indicates that the extraglandular microproliferation of argyrophil cells (IIIB) is a malignant lesion as a "microcarcinoid" at its very beginning of neoplastic formation in a form of "budding" as indicated in Fig. 3A–C in the article [2]. Such a developmental process of invasion prior to metastases is thought to be identical to the process in other organs not only of the other sites of the gastrointestinal tract, but also in other sites including the extradigestive organs.

### A comparison of metastasis rates in early stage: sm-lesions of carcinoids and ordinary carcinomas

Malignancy represented by metastasis rates in early stages with depth of invasion of the lesions restricted within the submucosa (sm-lesion) is discussed in a statistical comparison between two groups of carcinoid (n = 1158) and ordinary carcinoma (n = 1141) in Table 9 of the article [3].

In the stomach, the metastasis rates of the two groups of carcinoid versus ordinary carcinoma are calculated as 21.4% versus 3.1% in the size range category of 10.1 mm – 20.0 mm (p < 0.0001).

In the rectum, the metastasis rates of these two groups are described as 27.6% versus 10.0% in the same size category (p < 0.05), and as 32.4% versus 9.8% in the size range category over 10.1 mm as a whole (p < 0.0001). These results show that early stage carcinoids, with two prescribed factors of depth of invasion restricted within the submucosa and tumor size range of 1 cm to 2 cm, metastasize earlier than ordinary carcinomas with the identical description in both the stomach and rectum.

### Terminology

The term "carcinoid" has been deprecated by several authors mostly based on the metastatic potentiality (Table 2), as unfortunate [4], misleading and unsafe [5], outmoded [6,7], archaic [8,9], confusing [10], and even a misnomer by some authors [3,11-15]. One of the most adequate and brief terms for "carcinoid" that is included now in the group of GEP-NETs (gastroenteropancreatc neuroendocrine tumors) or simply NETs [8,16,17] would be "endocrinocarcinoma" [18-21], followed by NEC (neuroendocrinocarcinoma) or GEC (gut endocrinocarcinoma).

**Table 2:** the term "Carcinoid"

**Evaluation**	**Authors**	**Year**	**Reference**
Unfortunate	Willis RA	1940	[4]
Misleading	Roberts TW	1958	[5]
Outmoded	Wick MR, et al	1988	[6]
	Klemm KK, et al	1999	[7]
Archaic	Modlin IM, et al	1997	[8]
	Modlin IM	2005	[9]
Confusing	Andrés R	2002	[10]
Misnomer	Soga J	1973	[11]
	Rowe LD	1979	[12]
	Moertel CG	1987	[13]
	Soga J, et al	1999	[14]
	Soga J	2003	[15]
	Soga J	2005	[3]

On the other hand, since the term "carcinoid" has been so attractive and popularly used on a worldwide scale, and will be alive in the future for searching systems such as PubMed or Index Medicus, it would be very difficult and inconvenient to eliminate this term in a short period of time. Meanwhile this term and a newly accepted term, if decided, should be interchangeable with each other for the purpose of automated searching: for a concrete example, the new term with carcinoid in parentheses: [endocrinocarcinoma (carcinoid) ....].

Most important is that the term "carcinoid" should be used for a certain number of years, at least during the present generation of more or less 50 years, in the author's estimation, and be described without an adjective "benign" or "malignant" in recognition of the real entity of this particular malignant tumor group. Then, the necessity of the term "carcinoid" might be discussed by the next generation concerning its usefulness in automated searching for the literature.

No "benign" carcinoid without local invasion has been available up to this date either in the digestive organs or extradigestive sites in the author's experience.

Only complete serial sections of a seemingly encapsulated lesion could prove the benignancy, if any, with definite confirmation for the absence of a break of the capsule by microinvasion or budding. This would be, however, practically impossible. The histologic patterns or classification [11,14] would be still well applicable to "endocrinocarcinoma" as an initial morphologic implication for diagnosis.

The adequate term should be globally and historically discussed on several proposals along with future problems in relation to the real entity of this tumor group, considering the evaluation of the Consensus Conference [17].

### Changes in concepts of "carcinoid"

It is extraordinarily courageous to coin a new concept of tumor entity, as did Oberndorfer, a 31-year-old enthusiastic young scientist at that time in the year 1907 [1], and similarly to criticize a well-established and world-widely accepted concept introduced even in the textbooks. However, a change corrected on the basis of the truth is always required in science.

## Conclusion

The term "carcinoid" is a misnomer: the malignancy of this tumor group can be confirmed on the basis of local invasion prior to metastases. "No metastases without local invasion" is not of a negligible importance.

The adequate term should be globally and historically discussed in relation to the real entity of this tumor group, considering the evaluation of the Consensus Conference.

## Competing interests

The author has been retired from any institutional career for almost four years, and he has no competing interests of either a financial or a non-financial type in relation to this manuscript.

## Author's information

Recipient: (1) IRPC Eminent Scientist of the Year 2004: World Scientists Forum International Awards in Surgery and Surgical Pathology, 2004.

(2) ENETS Life Achievement Award and (3) IPSEN Oberndorfer Prize, at the 5^th^

ENETS in Paris, 2008.

IRPC: International Research Promoting Council.

ENETS: European Neuroendocrine Tumor Society.

IPSEN: Institut de Produits de Synthèse et d'Extraction Naturelle.

NET: Neuroendocrine Tumor/NEC: Neuroendocrine Carcinoma.
